# A cross-national comparison of violence among young men in China and the UK: psychiatric and cultural explanations

**DOI:** 10.1007/s00127-017-1420-y

**Published:** 2017-08-11

**Authors:** Jeremy Coid, Junmei Hu, Constantinos Kallis, Yuan Ping, Juying Zhang, Yueying Hu, Laura Bui, Simone Ullrich, Paul Bebbington

**Affiliations:** 1 Violence Prevention Research Unit, Wolfson Institute of Preventive Medicine, Queen Mary University of London, Barts and The London School of Medicine and Dentistry, Garrod Building, Turner Street, London, E1 2AD UK; 2 West China School of Basic Medical Sciences & Forensic Medicine, Sichuan University, Chengdu, 610041 China; 3 Chengdu Academy of Social Sciences, Chengdu, 610031 China; 4 West China School of Public Health, Sichuan University, Chengdu, 610041 China; 5 Division of Psychiatry, University College London, London, W1T 7NF UK; 6 Key Laboratory of Evidence Science, China University of Political Science and Law, Ministry of Education, Beijing, 102249 China

**Keywords:** Young men, Prevalence of violence, Cross-cultural differences, Explanatory variables

## Abstract

**Purpose:**

Public health psychiatry has a key role in violence prevention. Cross-national comparisons of violence and associated psychiatric morbidity can indicate targets for preventive interventions.

**Method:**

Data on young adult men in households, 18–34 years, were drawn from the Second Men’s Modern Lifestyles survey in Great Britain (*n* = 2046) and from a corresponding survey in Chengdu, China (*n* = 4132), using a translated questionnaire. Binary logistic regression models were carried out to estimate the cross-national differences for different types of violence and to identify explanatory variables.

**Results:**

Chinese men were less likely to report violence in the past 5 years (AOR 0.59, 95% CI 0.48–0.72, *P* < 0.001). All levels of violence were lower among Chinese men except intimate partner violence (AOR 2.43, 95% CI 1.65–3.59, *P* < 0.001) and a higher proportion of Chinese men were only violent towards their partners (AOR 7.90, 95% CI 3.27–19.07, *P* < 0.001).

**Conclusions:**

Cross-national differences were explained by British men’s reports of early violence persisting into adulthood, confidence in fighting ability, perception that violence is acceptable behaviour, and experience of violent victimization. More British men screened positive for antisocial personality disorder and substance misuse. Attitudes which condone violence and a serious problem of alcohol-related, male-on-male violence are key targets for preventive interventions among British men. The higher prevalence of life course-persistent antisocial behaviour among British men is of concern and requires further investigation. Higher prevalence of intimate partner violence among Chinese men reflects patriarchal approaches to conflict resolution and confirms an important public health problem in China which requires further cross-national investigation.

## Introduction

Violence is a global public health problem [1] and public health psychiatry has a key role in violence prevention [2]. Perpetration of serious violence and victimization involving serious injury in all countries disproportionately involves young men. Interventions for male-perpetrated violence benefit from being informed by prevalence estimates and national and cross-national comparisons of psychosocial and biological determinants, together with the role of cultural and other contextual factors. The comparison of countries with high and low rates of violence can be used to identify risk and protective factors and to develop preventive interventions.

China is among countries with low homicide rates, and the UK has a lower rate still (1.8 vs. 0.7 per 100,000 population aged 10–29 years [1]). However, non-lethal violence in the UK is more frequent than would be expected from its homicide rates. Convictions for robbery (2–3 per 1000 population) and common assault (3–4 per 1000 population) are similar to the USA [3], as are levels of self-reported violence (12%) [4, 5]. China has long been considered a low-crime country as a result of communist ideology and political directives [6–8]. Little was known about crime in China until the recent economic reform and open door policy [8]. Furthermore, information on self-reported crime including prevalence and associated factors is scarce [9] due to difficulty in collecting self-report data [10]. Official crime statistics for non-lethal violence in China are often unavailable at province or smaller unit levels and lack the measurement stability found in Western countries like the USA and UK [8]. Interest in gathering information to understand crime and violence in China has increased in the past two decades, but recent work has been primarily descriptive, and unsuitable for identification of patterns and trends [11].

The present study is a cross-national examination of self-reported violence in young adult men using representative cross-sectional surveys in the UK and Chengdu, China. The same measures of childhood maltreatment, psychiatric morbidity, and attitudes towards and experiences of violence were used to determine the explanations for differences and similarities between the two countries.

We aimed to: (1) compare the prevalence rates of violence among men; (2) identify and compare the correlates of violence; (3) account for the observed differences in prevalence; and (4) identify the implications for preventive interventions.

## Method

### Data collection

The survey in Great Britain was carried out in 2011 based on random location sampling. This advanced form of quota sampling reduces biases introduced when interviewers choose the locations to sample from. Individual sampling units (census areas of 150 households each) were randomly selected from British regions in proportion to their population to derive a representative sample of young men 18–34 years from England, Scotland, and Wales. The procedures have been described elsewhere in detail [12].

The survey in Chengdu, Sichuan Province, China, was carried out in two waves in 2012 and 2013 based on random location sampling as in the UK survey. Given the absence of a simple sampling frame, we applied a multi-stage stratified random sampling method. First, we stratified Greater Chengdu into three concentric rings, delineating (1) the city centre (exclusively urban “districts”); (2) suburbs (mixed rural “counties” and urban “districts”); and (3) rural areas (exclusively rural “counties”). Our sampling strategy varied according to concentric ring (strata) and administrative organization of households. All sampling frames were derived using official data provided by the Chengdu Government website.^1^

Informed consent was obtained from all survey participants and they were assured of confidentiality of the information obtained in the questionnaire. Respondents completed the pencil-and-paper questionnaire in private. They were paid £5 for participation in the British survey and given a gift to the value of 50 Yuan (approximately, £5) in Chengdu.

Weights were constructed for both surveys (RIM weighting in the UK survey; probability weighting in the Chinese survey) to ensure representativity of the sample. All descriptive and subsequent statistical comparisons were based on weighted data.

### Survey measures

A self-administered questionnaire developed/tested in Great Britain was translated into Mandarin. It was then independently back-translated into English to ensure equivalence with the English version [13].

The Psychosis Screening Questionnaire [14] was used to screen for psychosis. Participants were deemed screen positive if they met three plus criteria. Antisocial personality disorder (ASPD) was identified using the Structured Clinical Interview for DSM-IV Personality Disorders Screening Questionnaire [15].

Anxiety and depression were identified by a cutoff score of ≥11 in the past week on the Hospital Anxiety and Depression Scale [16]. The Alcohol Use Disorders Identification Test [17] was administered to identify hazardous drinking (scores ≥ 8), alcohol misuse (≥16), and alcohol dependence (≥20). A score of ≥6 on the Drug Use Disorders Identification Test (DUDIT) [18] indicated drug misuse disorder.

The PSQ has been successfully administered in a previous Chinese survey [19]. The psychometric properties of the HADS and AUDIT demonstrated good psychometric properties in Mandarin-speaking samples [20, 21]. With regard to the DUDIT, we followed the authors’ recommendations for translation.^2^

### Violence, violent attitudes, and child maltreatment

All participants were questioned about violent behaviour using questions from previous UK surveys [5]. They were asked whether they had “been in a physical fight, assaulted, or deliberately hit anyone in the past 5 years”. They were subsequently asked questions about the level of seriousness, number of incidents, intoxication, victims, and location of the violence. Furthermore, they were asked if they had been a victim of violence themselves.

A series of questions covered the young men’s attitudes towards violence: what they would do if threatened with a weapon, whether they had been brought up not to back down from a fight, had deliberately gone out looking for a fight, had ruminated about violence towards others, found violence exciting, had engaged in violence at sporting activities, had been involved in gang fights, or had carried a weapon in the past 5 years.

They were asked if they had witnessed parents or carers fighting in their home, had been subjected to physical or sexual abuse, or had been neglected before the age of 16.

### Statistical analysis

For descriptive purposes, weighted absolute/relative frequencies were reported for binary/polytomous variables and weighted means and standard deviations for variables on interval/ratio level.

In a first step, we investigated associations between demographic characteristics and nationality (British/Chinese). As survey weights were included to take into account the probability of selection in the sample, *F* tests were performed.

We carried out binary logistic regression models to estimate cross-national differences for different types of violence. We then estimated adjusted differences between Chinese and British young men for candidate explanatory variables for any violence from three domains: attitudes to and experiences of violence, child maltreatment, and psychiatric morbidity.

We performed binary logistic regression models to decide whether individual candidate variables might account for the differences in violence between British and Chinese men, based on the following criteria:

1. The association between violence and the candidate explanatory variable was statistically significant.
2. The association between the explanatory variable and nationality was statistically significant.
3. The relationship between nationality and violence was substantially attenuated after adjusting for the explanatory variable.

Attenuation in magnitude of the association between violence and nationality was quantified using the percentage explained by each candidate variable:

$$100 \times \frac{{\left( {\beta_{\text{base}} - \beta_{ \exp } } \right)}}{{\beta_{\text{base}} }},$$

where *β*_base_ and *β*_exp_ are the log-odds ratios for the cross-national difference in violence before and after adjusting for the candidate variable.

In each domain, explanatory variables meeting the above criteria were included in a binary logistic regression to test if their association with violence remained significant after adjusting for other variables from the same domain. Variables found not to be significant were excluded. The percentage of the baseline difference in violence explained by the variables included in the final model in each domain was also calculated using the above formula.

The final step was to combine explanatory variables from each final domain-specific model into a multivariate binary logistic regression model. Explanatory variables found not to be significantly associated with violence after adjusting for explanatory variables from other domains were excluded from the multivariate model. The final model included all significant explanatory variables from all domains. The percentage of the baseline difference was also calculated.

A significance level of 5% was adopted throughout. Analyses were carried out using Stata 14.

## Results

The cross-national comparison of 2046 British and 4132 Chinese men demonstrated that 622 (31.7%) and 901 (22.0%) participants, respectively, reported violence towards others in the past 5 years. The prevalence was significantly lower among Chinese men (AOR 0.59, 95% CI 0.48–0.72, *P* < 0.001).

The demographic characteristics of both samples are shown in Table 1. Significantly fewer Chinese men were single, more had higher educational qualifications, fewer were from ethnic minorities, and more were of lower occupational status. Their mean age was not significantly different when compared to British men. Comparing violent with non-violent men within countries, British violent men were more likely to be single, had fewer educational qualifications, fewer were from ethnic minority groups, and more were of lower educational status. These demographic characteristics corresponded to violent Chinese men, except for differences in educational qualifications and belonging to an ethnic minority. Finally, we compared Chinese violent men with British violent men and found that significantly more Chinese violent men had obtained higher educational qualifications, were less likely to be from ethnic minorities, and were younger.

**Table 1:** Demographic characteristics of British and Chinese men

	Comparison of British and Chinese men^a^						Comparison of British and Chinese violent men (subsample analyses)^b^								Comparison of violent men^a^	
	British (*n* = 2046)		Chinese (*n* = 4132)				British (*n* = 622)				Chinese (*n* = 901)					
	*n*	%	*n*	%	*F*	*P*	*n*	%	*F*	*P*	*n*	%	*F*	*P*	*F*	*P*
Single marital status	1212	59.7	2172	52.7	13.37	<0.001	414	67.1	20.26	<0.001	641	71.2	42.54	<0.001	1.26	0.261
Higher educational qualifications	432	21.9	1251	31.2	30.75	<0.001	85	14.1	29.66	<0.001	199	22.9	10.40	0.001	9.22	0.002
Ethnic minority group	246	12.1	109	2.6	124.88	<0.001	49	7.9	15.56	<0.001	33	3.8	1.96	0.161	7.57	0.006
Lower occupational status	1443	75.5	3029	79.8	7.53	0.006	476	81.6	16.36	<0.001	663	83.5	3.29	0.070	0.48	0.491

	*M*	SD	*M*	SD	*F*	*P*	*M*	SD	*F*	*P*	*M*	SD	*F*	*P*	*F*	*P*
Age	26.10	4.97	25.89	4.95	1.20	0.274	25.18	4.98	30.13	<0.001	23.97	4.61	47.71	<0.001	10.53	0.001

*N* and % reflect absolute and relative frequencies of independent variables among the two groups

^a^Reference group: British men

^b^Reference group: non-violent men

### Prevalence of violence

The prevalence of violent behaviour among British and Chinese men is reported in Table 2. After adjustment, fewer Chinese men reported any violence over the past 5 years, when intoxicated, repetitive violence, perpetrator and victim injury, police involvement, or minor violence. Chinese men were less likely to assault family members, persons known to them, strangers, and the police. They were also less likely to assault or get into fights with other persons in their own home, another person’s home, outdoors, or in a bar. However, Chinese men were more likely to assault intimate partners. Among those who committed intimate partner violence (IPV), 25.1% (*n* = 17) of the British men specialized in this form of violence (they did not engage in violent behaviours towards other victims) compared to 74.1% (*n* = 225) of the Chinese young men. This difference was statistically significant (AOR 7.90, 95% CI 3.27–19.07, *P* < 0.001).

**Table 2:** Comparison of Chinese and British men regarding violence, types of victims, and location

	British		Chinese		AOR	95% CI
	*n*	%	*n*	%		
Any violence	622	31.7	901	22.0	0.59***	0.48–0.72
Violent when intoxicated	336	17.3	50	1.2	0.05***	0.03–0.09
Repetitive violence	96	5.0	77	1.9	0.36***	0.22–0.60
Perpetrator injured	283	14.4	173	4.4	0.28***	0.19–0.40
Victim injured	311	15.8	376	9.5	0.58**	0.41–0.81
The police became involved	190	9.7	78	2.0	0.20***	0.12–0.33
Minor violence	161	8.2	238	6.0	0.71*	0.53–0.97
Victims						
Intimate partners	66	3.4	303	7.5	2.43***	1.65–3.59
Family member	78	3.9	59	1.5	0.37**	0.19–0.69
A friend	161	8.2	357	8.8	1.05	0.77–1.42
Someone known	205	10.4	70	1.7	0.13***	0.08–0.21
A stranger	356	18.1	71	1.8	0.09***	0.06–0.13
Police	55	2.8	13	0.3	0.12***	0.05–0.27
Other	40	2.1	42	1.0	0.65	0.32–1.32
Location						
Respondent’s home	81	4.1	60	1.5	0.38**	0.22–0.66
Someone else’s home	80	4.1	26	0.6	0.16***	0.09–0.28
In the street/outdoors	385	19.6	397	10.0	0.43***	0.31–0.60
In a bar or pub	286	14.5	97	2.4	0.13***	0.09–0.18
At the workplace	27	1.4	52	1.3	1.32	0.59–2.92
In a hospital	3	0.1	7	0.2	7.83	0.51–121.34
Anywhere else	88	4.5	129	3.2	0.73	0.50–1.07

Adjusted for higher educational qualifications, single marital status, ethnic minority, lower occupational class, and age. Reference group: British young men

*P < 0.05, **P < 0.01, ***P < 0.001

### Identification of explanatory variables

The comparison of British and Chinese young men’s attitudes towards and experiences of violence is shown in Table 3. Fewer Chinese men endorsed that they avoid violence and that they were brought up as a child not to back down from a fight. If someone threatened them with a weapon, Chinese young men were less likely to do nothing, more likely to run away, more likely to retaliate violently, and more likely to get a weapon and seek revenge. Significantly more Chinese men reported to easily lose their temper and become violent, to act violently when humiliated or disrespected, to actively look for a fight, having carried a knife, and been involved in gang fights. Compared to the British men, they were less likely to endorse doing better than average in a fight, to think about hurting other people, and having been violently victimized.

**Table 3:** Identification of explanatory variables: associations with nationality

	British		Chinese		AOR	95% CI
	*n*	%	*n*	%		
Attitudes towards violence						
Fear of violent victimization	289	14.1	581	14.5	1.14	0.91–1.42
Avoids violence	1325	64.8	2431	59.7	0.76**	0.64–0.90
Was taught not to back down from fight	714	34.9	881	21.7	0.49***	0.41–0.58
If threatened with weapon: would do nothing	630	30.8	582	14.5	0.38***	0.32–0.46
If threatened with weapon: would run away	789	38.5	2283	57.0	2.12***	1.80–2.49
If threatened with weapon: would retaliate violently	531	26.0	1795	44.7	2.20***	1.86–2.61
If threatened with weapon: would get a weapon	147	7.2	821	20.4	3.23***	2.53–4.12
Easily loses temper, becomes violent	214	10.4	716	17.7	1.76***	1.41–2.21
Believes to be better in a fist fight	617	30.1	845	20.7	0.53***	0.44–0.63
Violent ruminations	172	8.4	262	6.5	0.61**	0.45–0.83
Violent if disrespected	358	17.5	1181	29.0	1.88***	1.56–2.26
Feels remorse due to violent behaviour	291	14.2	606	14.9	0.97	0.79–1.20
Excited by violence	125	6.1	188	4.6	0.77	0.56–1.06
Has been actively looking for fights	79	3.9	264	6.5	1.74**	1.21–2.51
Instrumental violence	63	3.1	102	2.5	0.75	0.47–1.21
Carried a knife	106	5.2	479	11.8	2.41***	1.78–3.26
Violence at sporting events	114	5.6	211	5.2	0.82	0.60–1.11
Gang fights	56	2.8	425	10.5	3.69***	2.55–5.32
Victim of violence	335	16.4	454	11.4	0.63***	0.50–0.80
Childhood maltreatment						
Witnessing domestic violence	309	15.1	546	13.2	0.80	0.64–1.01
Sexual abuse/assault	48	2.4	30	0.8	0.43**	0.23–0.81
Physical abuse	125	6.1	66	1.7	0.26***	0.17–0.39
Neglect	78	3.8	640	15.9	4.90***	3.62–6.62
In care	77	3.9	65	1.6	0.38**	0.20–0.73
Psychiatric morbidities						
Psychosis	39	2.0	203	5.0	2.91***	1.85–4.57
Anxiety	212	10.7	376	9.2	0.96	0.73–1.26
Depression	151	7.6	560	13.8	2.31***	1.72–3.09
Hazardous drinking	894	47.1	1236	32.3	0.49***	0.41–0.59
Alcohol abuse	273	14.0	276	6.9	0.46***	0.37–0.59
Alcohol dependence	140	7.2	139	3.4	0.49***	0.36–0.68
Drug abuse	287	14.9	51	1.2	0.07***	0.05–0.11
Antisocial personality disorder	248	12.6	179	4.4	0.28***	0.21–0.37

Adjusted for higher educational qualifications, single marital status, ethnic minority, lower occupational class, and age. Reference group: British young men

*P < 0.05, **P < 0.01, ***P < 0.001

Chinese young men were less likely to have been subjected to sexual abuse or assault, physical abuse, and being brought up by carers during childhood but were more likely to report neglect.

Regarding psychiatric morbidity, Chinese men were at a significantly higher risk of screening positive for psychosis or depression. They were less likely to screen positive for hazardous drinking, alcohol abuse, alcohol dependence, drug abuse, and ASPD.

With regard to the consumption of alcohol, a substantially larger number of Chinese men were abstainers (1297, 33.1% vs. 226, 11.7%; AOR 4.70, 95% CI 3.82–5.78, *P* < 0.001), and their AUDIT scores were positively correlated with age (AOR 1.04, 95% CI 1.01–1.07, *P* = 0.023).

In a second step, we tested which of these potentially explanatory variables examined in Table 3 demonstrated a statistically significant relationship with any violence. Following adjustments, a significant association was found with worries of becoming a victim of violence, having been brought up as a child not to back down from a fight, retaliate violently when threatened with a weapon or getting a weapon and seeking revenge, easily losing temper and acting violently, doing better than anyone else in a fist fight, thinking about hurting other people, acting violently when disrespected, remorseful about violent behaviour, fighting because of excitement, actively looking for a fight, using violence to get what they wanted, carrying a knife, being involved in violence in sporting events, being involved in gang fights, and violent victimization. Inversely related was doing nothing if someone threatened them with violence (Table 4). As can be seen in Table 4, most childhood maltreatment variables were significantly associated with any violence apart from neglect and having been in care, and all psychiatric morbidities demonstrated a relationship, with depression being inversely associated.

**Table 4:** Identification of explanatory variables: associations with any violence in the total combined sample (British and Chinese men)

	AOR	95% CI
Attitudes towards violence		
Fear of violent victimization	1.48**	1.14–1.91
Avoids violence	0.66***	0.53–0.82
Was taught not to back down from fight	2.94***	2.38–3.64
If threatened with weapon: would do nothing	0.75*	0.60–0.95
If threatened with weapon: would run away	0.82	0.66–1.02
If threatened with weapon: would retaliate violently	3.17***	2.57–3.93
If threatened with weapon: would get a weapon	3.44***	2.66–4.46
Easily loses temper, becomes violent	3.49***	2.69–4.52
Believes to be better in a fist fight	4.54***	3.63–5.67
Violent ruminations	4.72***	3.39–6.57
Violent if disrespected	3.28***	2.63–4.10
Feels remorse due to violent behaviour	6.25***	4.89–7.99
Excited by violence	8.03***	5.54–11.66
Has been actively looking for fights	9.51***	6.11–14.80
Instrumental violence	5.93***	3.38–10.42
Carried a knife	3.94***	2.86–5.44
Violence at sporting events	4.06***	2.82–5.83
Gang fights	5.94***	3.93–8.97
Victim of violence	4.20***	3.27–5.39
Childhood maltreatment		
Witnessing domestic violence	1.77***	1.38–2.29
Sexual abuse/assault	2.32**	1.31–4.09
Physical abuse	2.74***	1.90–3.93
Neglect	1.24	0.92–1.68
In care	2.10	0.89–4.95
Psychiatric morbidities		
Psychosis	2.79***	1.74–4.48
Anxiety	1.98***	1.47–2.67
Depression	0.60*	0.41–0.89
Hazardous drinking	2.95***	2.38–3.65
Alcohol abuse	2.76***	2.14–3.56
Alcohol dependence	2.87***	2.04–4.03
Drug abuse	5.10***	3.83–6.79
Antisocial personality disorder	9.92***	7.15–13.76

Adjusted for higher educational qualifications, single marital status, ethnic minority, lower occupational class, and age

*P < 0.05, **P < 0.01, ***P < 0.001

### Explaining cross-national differences in violence

Variables which were significantly associated with both nationality and any violence (Tables 3, 4) were considered explanatory variables in subsequent analyses. The baseline model testing the association between nationality and any violence (Table 2) was then extended by adjusting for these explanatory variables (separately). The adjusted ORs are reported in Table 5 and % change reflects change in magnitude of the effect compared to the baseline model. Only positive % change was considered relevant since they reflected an increase in ORs implying that the differences between Chinese and British young men were getting smaller. With regard to attitudes towards violence, four variables were considered relevant including: was taught not to back down from fight, believes to be better in a fist fight, violent ruminations, and violent victimization. When entered simultaneously in the statistical model, the OR of the association between nationality and any violence was 0.77 (95% CI 0.61–0.98, *P* = 0.032) and % change was 51.8%. Physical abuse was the only variable in the childhood maltreatment domain which decreased the difference between British and Chinese men regarding violence. In the psychiatric morbidity domain, all mental disorders except psychosis explained to some extent the differences between Chinese and British men. When entered simultaneously, the OR of the association with violence was 0.95 (95% CI 0.74–1.19, *P* = 0.599) with % change of 88.1%.

**Table 5:** Explanatory variables for the association between nationality (Chinese) and with any violence

	AOR	95% CI	% Change
Baseline model			
Chinese vs. British (reference group) young men	0.59***	0.48–0.72	
Explanatory variables			
Attitudes towards violence			
Avoids violence	0.57***	0.46–0.69	−7.3
Was taught not to back down from fight	0.68***	0.55–0.84	28.3
If threatened with weapon: would do nothing	0.54***	0.44–0.67	−14.5
If threatened with weapon: would retaliate violently	0.44***	0.36–0.55	−53.5
If threatened with weapon: would get a weapon	0.46***	0.36–0.58	−48.0
Easily loses temper, becomes violent	0.52***	0.41–0.65	−23.1
Believes to be better in a fist fight	0.70**	0.56–0.87	32.3
Violent ruminations	0.61***	0.49–0.75	6.0
Violent if disrespected	0.48***	0.38–0.61	−36.6
Has been actively looking for fights	0.52***	0.42–0.65	−23.2
Carried a knife	0.50***	0.40–0.63	−28.9
Gang fights	0.48***	0.38–0.60	−37.7
Victim of violence	0.61***	0.49–0.76	7.1
Childhood maltreatment			
Sexual abuse/assault	0.58***	0.47–0.71	−2.5
Physical abuse	0.60***	0.49–0.74	4.0
Psychiatric morbidities			
Psychosis	0.55***	0.44–0.68	−12.8
Depression	0.6***	0.49–0.74	3.8
Hazardous drinking	0.73**	0.59–0.90	41.0
Alcohol abuse	0.64***	0.52–0.79	15.0
Alcohol dependence	0.61***	0.50–0.75	6.9
Drug abuse	0.74**	0.59–0.91	42.5
Antisocial personality disorder	0.73**	0.59–0.91	40.3

Adjusted for higher educational qualifications, single marital status, ethnic minority, lower occupational class, and age

Reference group: *P < 0.05, **P < 0.01, ***P < 0.001

In a final model, all relevant domain variables were entered together resulting in an OR of 1.09 (95% CI 0.84–1.42, *P* = 0.522) and the cross-national baseline difference in violence was fully explained by these variables (116.2%).

## Discussion

Chinese men were less likely than British men to report all forms of violence except IPV. Following adjustment, the odds of Chinese men reporting any violence was substantially lower than that of British men. All levels of seriousness were less prevalent. These differences were accounted for by higher levels of ASPD and substance misuse among British men, together with their greater willingness to confront an aggressor, confidence in their fighting ability, violent ruminations, and previous experience of violent victimization.

Although the perpetration of IPV was more often reported by Chinese men, violent incidents in the home (where IPV is more likely to occur) were more commonly reported by British men, together with violence in the homes of others, in bars/pubs, and outdoors. This corresponded to the wider range of victims reported by British men. Outdoor settings were the most commonly reported in both countries. However, the most frequent victims of the British participants were acquaintances and strangers, while the most frequent among Chinese men were their friends. Nevertheless, there was no significant national difference in the rates of violence towards friends.

The largest difference observed was for incidents in which the perpetrator admitted intoxication with alcohol and/or drugs. Taken together with the higher rates in British men of violence outdoors, in bars/pubs, with acquaintances or strangers, and of victim or perpetrator injury, our findings confirmed a major problem of alcohol-related violence among young British men.

Approximately, half of all violence in England and Wales is thought to be committed by persons under the influence of alcohol, in or around pubs, bars, or nightclubs [22]. Alcohol-related crimes cost the UK government £12 billion annually [23]. Corresponding hospital emergency department data also show that assault-related attendances are commonest at weekend nights, and that a large proportion of assaulted patients are young men who have been drinking in bars and nightclubs [24, 25]. Alcohol has a dose–response relationship with violence and with risk of violent injury [26–28]. The high prevalence of violence when intoxicated in British men also corresponds to greater acceptance of drunkenness in northern than in southern European countries. However, fighting when drunk is more common in British than in either German or Spanish men [29–31]. Alcohol use is an accepted part of Chinese culture and three-quarters of adult men consume alcohol; consumption differs according to age, gender, and region [32]. This was confirmed by our survey which showed that approximately one-third of Chinese men were alcohol abstainers (compared to 12% of British men) and their AUDIT scores were associated with increasing age. Traditional views in China condemn heavy drinking [33], and adverse effects of behaviour associated with drinking can increase risk of harmful outcomes [34, 35].

### Risk factors for violence

We confirmed that drug/alcohol misuse and ASPD were significantly more common in British men. These factors are strongly related to violence in the UK [5, 36] and were unsurprisingly retained in the final model, in which ASPD was most strongly associated with violence.

Conduct disorder before 15 years was a qualifying factor for the diagnosis of ASPD in this study. Many men with ASPD were therefore early-onset delinquents whose antisocial and aggressive behaviour had persisted into adulthood [37]. ASPD is strongly associated with violence [5, 38, 39], although it is partly defined on the basis of previous violent behaviour. While ASPD is less common in Taiwan than in the USA [40], Hong Kong [41] and South Korea [42] have relatively high rates, possibly due to higher rates of alcoholism in those two east Asian countries [43, 44]. Moffitt [37] proposed that early and persistent violent/criminal behaviour has its origins in neurological deficits and exposure to environmental risks, such as poor parenting and parental antisocial behaviour. However, child maltreatment did not explain cross-national differences in our study.

China has internationally low rates of illicit drug use [45], lower than other Asian and Pacific region countries [46]. However, China has more recently become a major producer of methamphetamines and their constituents for methamphetamine production in neighbouring countries [47–49]. Illicit drug use by men in Chengdu was very infrequent, and they did not favour any particular substance. In contrast, young British men overwhelmingly reported misuse of cannabis. The associations between substance misuse and violence have been debated [50]. Drugs and alcohol may cause violence through psychopharmacological properties, economic motivation to get drugs, or the systemic violence associated with illegal drug markets [51]. Alternatively, aggressive individuals may use substances such as cannabis for their calming effect. However, they may also actively seek situations involving heavy substance use to increase their levels of excitement through risk-taking, including violence [50].

The associations between violent experiences and attitudes towards violence were complex. Chinese men were less likely to report violence towards others, but more likely to report high-risk behaviours such as carrying a knife, deliberately going looking for a fight, and involvement in gang fights. They were more likely to behave violently if disrespected, signifying the importance of “loss of face” within Chinese culture. Despite commonly held notions of violence among British soccer spectators, there was no difference in the prevalence reporting violence at sporting events. Chinese men also reported they would be less likely to comply if an aggressor threatened them with a weapon, more likely to retaliate violently, and more likely to get a weapon and come back for the aggressor later. However, some Chinese men also reported that they would be more likely to run away. It is probable that these somewhat contradictory associations are explained by the fact that more British men had actual previous experience of taking part in violence, from a younger age, particularly those with ASPD. In addition, more had been violently victimized. The questions typically endorsed by Chinese men concerned attitudes conducive to violence and were hypothetical rather than actual situations. British men were more likely to report they had been encouraged in childhood not to back down from a fight, they had greater confidence in their fighting prowess, and more had been victims of violence.

The excessive violence in British men was associated with a number of factors reflecting the persistence of aggressive behaviour from childhood to adulthood, including encouragement from carers during childhood to see violence as an acceptable way of resolving disputes, and a lifestyle in adulthood where fighting is common, skills in fighting are highly regarded, in line with macho attitudes, and where violent victimization is a common and acceptable risk. Disinhibiting factors of intoxication in high-risk social environments associated with substance misuse, primarily alcohol, together with cultural acceptability and expectations of behaviour when intoxicated are also characteristic of male-on-male violence among British men. Such factors should be targeted among British men if the aim is to reduce their levels of violence.

### Limitations

Our study has several limitations. Although we carried out representative surveys in both countries, the Chinese survey was geographically restricted, to an area of west central China, and it was not possible to obtain sufficient Chinese census data to establish representativity with regard to other Chinese regions. However, we believe that the information we obtained to create weights ensured that the weighted sample was representative of the population of interest. Furthermore, the community-based design avoided the selection bias associated with clinical samples and the large samples provided sufficient statistical power to test complex models and to control for confounding from demographic characteristics and psychiatric morbidity.

We utilized the 2001 British census data (the survey of British young men was designed based on the 2001 census data) to investigate whether there were statistically significant differences compared to our survey with respect to age distribution and region. No such differences were found, a result which indicates the representativeness of our British survey of young men.

Violence was assessed by self-report: we lacked corroborative information, and participants may have been reticent about socially undesirable behaviours. The reporting of drug misuse in China may have been inhibited by penalties for manufacture and trafficking, which include capital punishment. However, treatment for drug dependence in China is widely available and does not include legal sanctions on the basis of addiction.

Diagnoses were derived from self-report questionnaires and not confirmed by clinical interview. However, self-report can compare favourably with clinician assessments [52], and the prevalence of mental disorders among young men in two previous surveys in Great Britain [53, 54] was similar to those of non-violent men in our British survey. There was careful attention to the translation of questions into Mandarin, and Chinese clinicians piloted their use and were involved in translation and back-translation. However, whilst actual behaviours and experiences may have been reported similarly, the connotations of certain questions regarding attitudes may have been influenced by cultural differences, leading to difficulties in translation. Furthermore, it could be argued that criteria of mental disorders differed between the UK and China. However, introduction of the third version of the Chinese Classification of Mental Disorders has led towards a substantial integration in the international classification of mental illness with strong similarities to Western classifications of mental disorders assessed in this survey [55].

### Implications

Public health policies targeting young problem drinkers in the UK, particularly 18- to 24-year-olds, are considered an urgent priority in reducing antisocial behaviour [56, 57]. Recommendations typically involve increasing prices, which are known to influence the purchase of alcohol in this age group [58], reducing availability of alcohol by reducing the density of outlets, and improving the atmosphere and behaviour in drinking venues through improved management by bar staff [59]. Alcohol is similarly available in shops and restaurants in China and comparatively affordable. However, binge drinking and pre-loading with alcohol before going to licensed premises [31], strongly associated with antisocial behaviour in the UK, are unusual among young Chinese drinkers. Alcohol consumption in bars is a relatively new phenomenon associated with Westernization. Alcohol consumption in restaurants has also increased, associated with increasing affluence through economic development, but is not a drinking behaviour typically associated with antisocial behaviour in China or the UK [60, 61]. Our findings indicate that, to be effective, these interventions need to be accompanied by others targeting more difficult areas: changing the acceptability of public drunkenness [62] and the cultural and social norms of behaviour associated with drinking behaviour in the UK.

Treatment programmes for conduct disorder have shown moderate benefits for future antisocial disorder. These aim to change the environment around young persons, with training for parents and carers, with multisystem therapy being the most effective component [63, 64]. However, such selective prevention requires accurate identification of individuals or subgroups of the population at risk of developing conduct disorder. Further cross-national research into why childhood antisocial disorder in the UK is much more likely to persist into adulthood than in China might lead to further developments in screening and intervention.

Domestic violence in China, particularly spousal abuse, has gained increasing attention due to a number of recent high-profile cases. Several Chinese surveys demonstrated that prevalence rates of domestic violence range between 30 and 40% [65, 66], but there is some evidence that the proportion could be significantly higher, especially in rural areas. This has led to a draft of a first Chinese national law against domestic violence published in November 2014 and promulgated in late 2915. In this draft, domestic violence was defined for the first time and referred to physical, psychological or other infractions committed between family members including spouses, parents, children, and other close relatives.^3^ A comparison of the prevalence of violence of men who were uniquely violent towards their partners showed that the numbers of IPV specialists were significantly higher among the Chinese men where approximately two-thirds of those who had committed intimate partner violence were violent only towards their partners. Only a quarter of the British young men who had committed IPV demonstrated this pattern.

The survey questions were directed primarily at male-on-male violence because it is the largest international public health problem of violence. A previous UK study of associations with psychiatric morbidity indicated the importance of ASPD: IPV was one of multiple forms of violence associated with this condition [5]. However, IPV in Chengdu was not particularly associated with ASPD. Furthermore, the percentage of men who were exclusively violent towards their partners in the UK was very small, suggesting that young men in this age range who are violent to partners are generally violent men. This would in turn suggest that treatment interventions aimed only at IPV and ignoring a generalized propensity to violence among UK men are unlikely to be effective. However, among Chinese men, such interventions, if focused on those who are only violent towards partners, may be appropriate. Further research should investigate Chinese men’s attitudes towards women’s roles, the acceptability of violence within relationships, and the need for intervention in IPV in China. There is evidence from clinical samples that IPV may also be more frequent in China than in the USA and other Western countries. This is thought to be associated with patriarchal values [67, 68] and traditional approaches to conflict resolution, where violence against women is generally concealed and protected within the area of private life, and tolerated or ignored [66]. Our findings therefore suggest marked contrasts between China and the UK in the patterns of violent behaviour that are both tolerated and condoned.

## Footnotes

1
http://jcpt.chengdu.gov.cn/chengdushi/.

2
http://www.emcdda.europa.eu/best-practice/eib/dudit.

3
http://chinalawtranslate.com/domestic-violence-law/?lang=en.
